# Coronary microvascular dysfunction in pregnancy: time to pay closer attention?

**DOI:** 10.3389/fcvm.2026.1734077

**Published:** 2026-02-24

**Authors:** Eirini Beneki, Nikolaos Pyrpyris, Athanasios Sakalidis, Eirini Dri, Panayiotis Iliakis, Theodoros Mprotsis, Francesco Perone, Aggelos Papanikolaou, Konstantinos Aznaouridis, Kyriakos Dimitriadis, Konstantinos Tsioufis, Constantina Aggeli

**Affiliations:** 1 First Cardiology Department, School of Medicine, Hippokration General Hospital, National and Kapodistrian University of Athens, Athens, Greece; 2 Department of Cardiology, Lausanne University Hospital, Lausanne, Switzerland; 3 Department of Biomathematics, School of Medicine, University of Thessaly, Larissa, Greece; 4 Cardiac Rehabilitation Unit, Rehabilitation Clinic ‘Villa delle Magnolie’, Caserta, Castel Morrone, Italy

**Keywords:** adverse pregnancy outcomes, cardiac magnetic resonance imaging, cardiovascular imaging, coronary microvascular dysfunction, echocardiography, gestational diabetes, hypertensive disorders of pregnancy, preeclampsia

## Abstract

Coronary microvascular dysfunction (CMD) is increasingly recognized as a significant cardiovascular condition, particularly among women, yet its diagnosis and management during pregnancy remain poorly understood. CMD may arise *de novo* in the context of hypertensive disorders of pregnancy or represent an exacerbation of pre-existing endothelial dysfunction. This article views current evidence surrounding CMD in pregnancy, outlines the limitations of current diagnostic and treatment approaches, and highlights critical research gaps that must be addressed to improve outcomes in this vulnerable population.

## Introduction

Coronary microvascular dysfunction (CMD) characterized by impaired coronary flow reserve (CFR) and endothelial dysfunction in the absence of obstructive coronary artery disease (CAD) is increasingly recognized as a significant cardiovascular condition in women, including during the peripartum period. Despite increasing recognition, treatment options for CMD during pregnancy remain undefined. The presence of hypertensive disorders such as preeclampsia may further exacerbate microvascular injury, potentially leading to persistent subclinical or symptomatic ischemia. Understanding the unique pathophysiology, the diagnostic limitations and therapeutic challenges of CMD during pregnancy is essential for early intervention and long-term cardiovascular risk mitigation. In this context, contemporary diagnostic strategies increasingly emphasize a comprehensive invasive functional assessment to characterize the underlying pathophysiology in patients with ischemia and no obstructive coronary arteries (INOCA), angina with no obstructive coronary arteries (ANOCA), and myocardial infarction with non-obstructive coronary arteries (MINOCA). The so-called “Full Physiology” approach, incorporating coronary pressure and flow measurements, vasoreactivity testing, and microvascular resistance assessment, enables precise endotyping of coronary vasomotor disorders and guides targeted therapy. This integrated strategy has recently been highlighted as a key step toward achieving a definitive diagnosis in these complex clinical syndromes (1).

**Figure 1:**
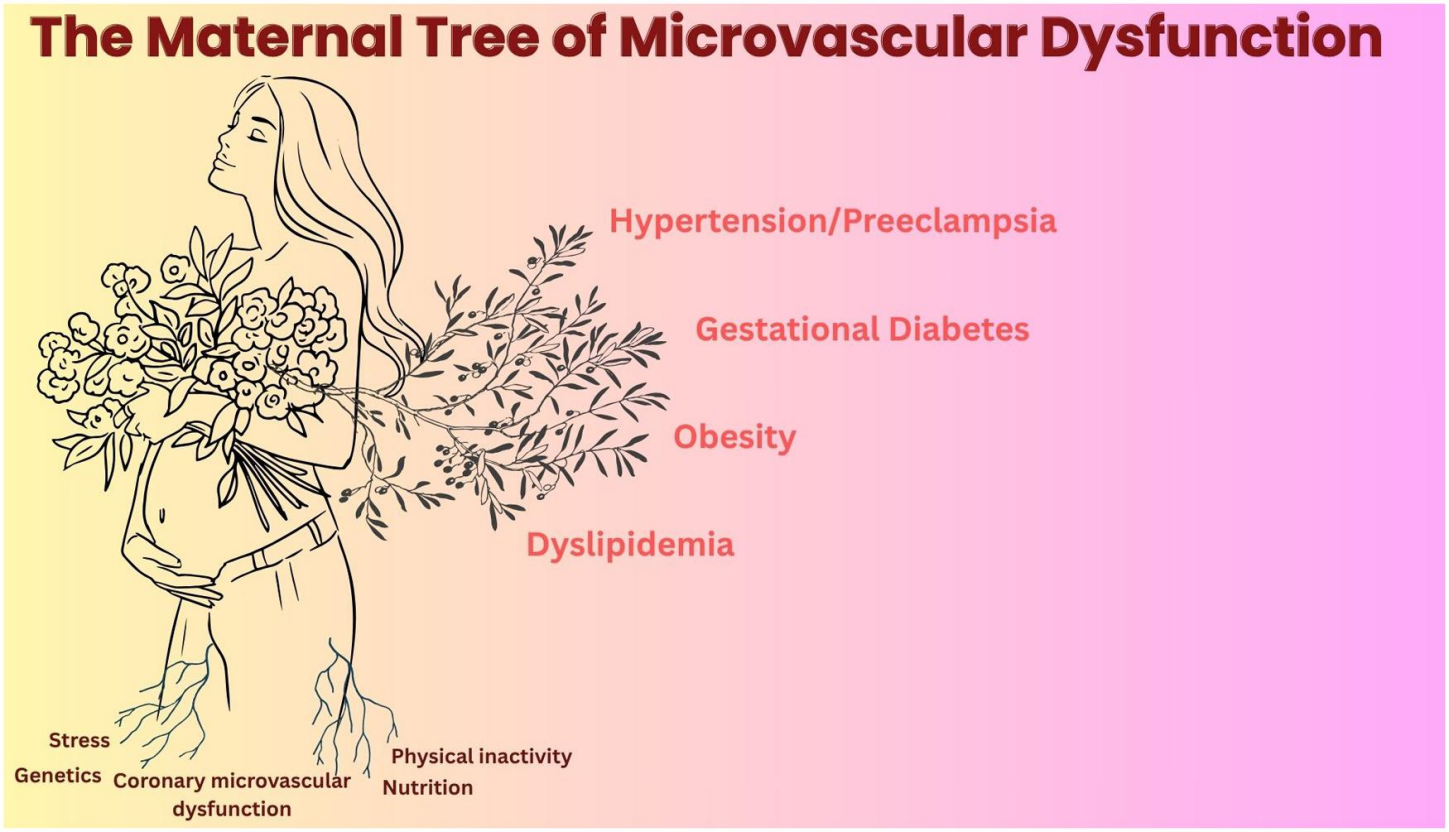
Maternity and microvascular dysfunction: The plethora of traditional and non-traditional risk factors increasing the odds for microvascular dysfunction and adverse cardiovascular events in women.

Beyond its established role in angina and ischemia in the absence of obstructive CAD (INOCA), CMD appears to have implications for reproductive health, including fertility and pregnancy outcomes. From another perspective, there is a growing degree of evidence that women who have suffered adverse pregnancy outcomes (APOs) such as preeclampsia and low birth weight are at an elevated risk for adverse cardiovascular outcomes later in life (2). However, the underlying mechanisms for this increased risk remain unclear (Figure 1).

## Search strategy and study selection

A comprehensive literature search was conducted in PubMed, Scopus and Web of Science to identify relevant studies published until September 2025. The search strategy included the terms “coronary microvascular dysfunction”, “pregnancy”, “hypertensive disorders of pregnancy”, “preeclampsia”, “gestational diabetes”, “adverse pregnancy outcomes”, “cardiovascular imaging”, “echocardiography” and “cardiac magnetic resonance imaging”. Only articles published in English were considered. Priority was given to original clinical studies and high-quality review articles focusing on pregnancy and cardiovascular imaging findings in the context of CMD; however, case reports were also screened due to the relative scarcity of available data. Additional relevant articles were identified through manual screening of reference lists. Conference abstracts were excluded.

## Endothelial dysfunction: the culprit mechanism?

Pregnancy is a physiological state associated with many complex hemodynamic, metabolic and humoral disturbances (3). In normal pregnancy, the increased production and responsiveness to vasodilators, and decreased sensitivity to vasoconstrictive hormones (4) leads to an increased vasodilatory tone and decreased maternal vascular resistance. These changes result in decreased blood pressure and increased sympathetic activation, maternal blood volume and cardiac output (5).

Disruption of these adaptive mechanisms, particularly at the level of the endothelium and microcirculation, has been proposed as a potential shared pathophysiological pathway linking APOs with future cardiovascular disease. However, this concept is primarily supported by observational data and causality has not been definitively established. Such vascular dysfunction may emerge early in pregnancy (or perhaps even before implantation), and persist or reappear later in life, contributing to long-term cardiovascular risk. Observational clinical evidence supporting this association derives from the Women's Ischemia Syndrome Evaluation—Coronary Vascular Dysfunction (WISE-CVD) study. In a subgroup of women with signs and symptoms of ischemia but no obstructive coronary artery disease (CAD), those with a history of APOs demonstrated significantly reduced CFR, a key marker of CMD compared to those without APOs (6). While these findings suggest that microvascular dysfunction may not only be a consequence of pregnancy complications but also a persistent marker of vascular vulnerability in women, they remain hypothesis -generating and warrant confirmation in larger prospective studies.

Shared pathophysiological mechanisms between cardiovascular and placental dysfunction may help explain this association. A key player is vascular endothelial growth factor (VEGF), a critical proangiogenic molecule necessary for both placental vascular development and endothelial hemostasis (7).

In conditions like preeclampsia, observational data indicate that VEGF signaling is disrupted, often due to elevated levels of its endogenous inhibitor, soluble fms-like tyrosine kinase-1 (sFlt-1), leading to endothelial dysfunction, impaired vasodilation, and systemic inflammation (8). These abnormalities resemble key features of CMD; however, the extent to which a shared angiogenic imbalance directly links placental and coronary microvascular pathology remains largely speculative and is primarily supported by observational data. These abnormalities mirror those seen in CMD, suggesting that similar mechanisms, angiogenic imbalance, inflammation, dysregulated vasoconstriction/dilation, and impaired microvascular function may underlie both placental and cardiac complications.

## CMD in pregnancy and preeclampsia: a missing link in women's cardiovascular risk

Based on this, recent observational studies have increasingly focused on CMD during pregnancy as a potential mechanism linking APOs with future cardiovascular disease. Preeclampsia, a hypertensive disorder of pregnancy marked by endothelial dysfunction and systemic inflammation, has been consistently associated in large epidemiologic cohorts with an increased long-term risk of cardiovascular disease, including heart failure with preserved ejection fraction (HFpEF) (9). Despite these associations, the direct impact of preeclampsia on peripartum coronary microvascular function has not yet been clearly delineated. To date, no studies have prospectively evaluated coronary microvascular function during the peripartum period in women with preeclampsia, leaving a critical gap in understanding the temporal evolution of CMD and its immediate and long-term consequences. Investigating this relationship could provide valuable insights into early subclinical cardiovascular changes in this high-risk population and may inform strategies for risk stratification, monitoring, and early intervention to prevent heart failure and ischemic heart disease in women with a history of preeclampsia.

## Fertility challenges in women with CMD

CMD is increasingly recognized not only as a cardiovascular condition but also as a contributor to reproductive health challenges in women of childbearing age. Evidence suggests that CMD may be associated with reduced fertility rates, although the relationship is likely multifactorial. The lower fertility observed in this population may reflect the underlying severity of vascular dysfunction, but also could be influenced by personal decisions to delay or avoid pregnancy, especially when symptom control is achieved through potentially teratogenic medications. Pharmacologic therapies commonly used in CMD management, including angiotensin-converting enzyme (ACE) inhibitors, angiotensin receptor blockers (ARBs), statins, and beta-blockers, carry teratogenic potential and have been associated with fetal complications such as growth restriction and teratogenicity (10). As a result, both clinicians and patients may adopt a cautious approach toward pregnancy planning, leading to underreported or delayed fertility attempts in this group.

Despite these concerns, real-world data remain limited. In a case series of women with CMD, only one of five women was using contraception and experienced an unplanned pregnancy, highlighting potential gaps in reproductive counseling and family planning (11). In contrast, previous studies have reported higher rates of contraceptive use among women with INOCA (44%) compared to the general population (29%), suggesting increased awareness and proactive planning among women with known vascular conditions (12, 13).

Taken together, these findings underscore the importance of multidisciplinary care involving cardiologists, obstetricians, and reproductive specialists to ensure that women with CMD receive individualized preconception counseling, risk assessment, and medication management. Greater attention to this intersection may help address the reproductive needs and preferences of this often-overlooked group.

## Adverse pregnancy outcomes and CMD

Observational data and evidence from a large epidemiologic cohort indicate that women with CMD have a higher incidence of APOs, including spontaneous miscarriage, fetal growth restriction, and preterm birth, compared to the general population (14, 15). Whether this elevated risk reflects the severity of underlying CMD, or broader systemic vascular dysfunction, remains an area of ongoing investigation.

A key insight into this relationship lies in the anatomical and physiological parallels between coronary and uterine microcirculations. The spiral arterioles supplying the endometrium that share striking structural and functional similarities with the coronary microvasculature. Both vascular beds are highly responsive to hormonal and metabolic cues and both rely on intact vasoactive signaling pathways, particularly the L-arginine-nitric oxide (NO) pathway, to maintain appropriate vasodilation and tissue perfusion (16). In CMD, impaired vasodilatory capacity and endothelial dysfunction, often due to reduced NO bioavailability, are central features. Importantly, this dysfunction is not necessarily confined to the coronary circulation. Disruption of NO signaling may also extend to the uteroplacental circulation, contributing to inadequate spiral artery remodeling and abnormal placental development, pathophysiological hallmarks of complications such as preeclampsia and fetal growth restriction.

In animal models, genetic deletion of endothelial NO synthase was associated with reduced uterine blood flow, impaired spiral artery elongation, and compromised placental oxygenation, directly linking NO deficiency to reproductive vascular dysfunction (17).

Although direct human evidence remains limited, these preclinical findings suggest that CMD may act as a surrogate marker for systemic microvascular dysfunction, including in the uterine vasculature. This perspective aligns with a growing recognition that microvascular pathology is not organ-specific but rather reflects a broader state of vascular vulnerability. Further evidence supporting this hypothesis includes the demonstrated association between CMD and renal microvascular dysfunction.

In women with signs and symptoms of ischemia, CMD has been linked to impaired renal perfusion and endothelial abnormalities reinforcing the concept of multi-organ microvascular involvement (18). The convergence of coronary, uterine, and renal microvascular abnormalities points toward a shared pathophysiological substrate, possibly driven by systemic endothelial dysfunction, oxidative stress, and chronic low-grade inflammation. These mechanisms may underlie the increased incidence of APOs in women with CMD and contribute to the subsequent elevation in long-term cardiovascular risk.

## Diagnostic approach strategy for CMD in pregnant women

Accurate diagnosis of CMD remains a clinical challenge, particularly in women with of reproductive age or those with a history of APOs. Careful consideration should be given to the selection of diagnostic modalities, as commonly used tests may not fully capture the spectrum of CMD, especially in early stages or in cases driven by endothelial dysfunction, which plays a central role in both CMD and pregnancy-related vascular complications.

Dipyridamole stress echocardiography, a widely available and non-invasive method, assesses CFR predominantly in the left anterior descending artery. However, it primarily evaluates endothelial-independent microvascular function and may not be sensitive to subtle endothelial abnormalities, particularly those relevant in conditions such as preeclampsia or early CMD. Supporting this limitation, a study from the iPOWER cohort found no significant association between a history of miscarriages or APOs and CMD as assessed by dipyridamole stress echocardiography (19). This apparent lack of association may therefore reflect methodological limitations related to CMD endotyping, rather than a true absence of microvascular disease. Accordingly, these findings suggest that dipyridamole stress echocardiography may underestimate CMD in populations where endothelial-dependent dysfunction predominates.

In contrast, emerging evidence emphasizes the relevance of endothelial-dependent CMD in mediating APOs, including preeclampsia. Impaired coronary flow reserve and PET-derived myocardial flow reserve have been consistently demonstrated in women with prior APOs in the WISE-CVD study and PET-based investigations, as well as in more recent hypertensive disorders of pregnancy cohorts (6, 8, 20).

In addition, maternal endothelial dysfunction has been directly associated with preeclampsia in observational studies, indicating that diagnostic tools capable of detecting endothelial dysfunction are critical in this context (21). The distinction between endothelial-dependent and endothelial-independent CMD likely explains, at least in part, the discrepant results observed across different imaging modalities and cohorts.

The gold standard for comprehensive CMD evaluation remains invasive coronary reactivity testing, which utilizes pharmacologic agents such as acetylcholine to assess endothelial-dependent vasodilation, and adenosine to evaluate endothelial-independent responses. These invasive tests offer precise physiological assessment of both epicardial and microvascular function, but their use is limited by procedural risk, cost and availability, factors that can restrict widespread application, especially in young or reproductive-age women (22).

Among non-invasive techniques, cardiac positron emission tomography (PET) has emerged as the most validated and reproducible tool for CMD assessment. PET allows for quantification of myocardial flow reserve (MFR), defined as the ratio of hyperemic to resting myocardial blood flow (MBF), which reflects the combined effects of epicardial and microvascular coronary function. In the absence of obstructive epicardial CAD, impaired MFR serves as a reliable marker of CMD. MFR measured via PET has strong prognostic value, predicting future major adverse cardiovascular events and heart failure with HFpEF in midlife and older adults (23, 24).

However, PET imaging is not without limitations, including cost, radiation exposure, and limited access in certain healthcare settings. As such, discrepancies between modalities are common, and the choice of diagnostic tool should be guided by the specific clinical context, availability and the suspected underlying pathophysiological mechanism. This lack of standardization presents a challenge for both research comparisons and clinical decision-making in women with suspected CMD and prior APOs.

## Management strategies for CMD during pregnancy

**Figure 2:**
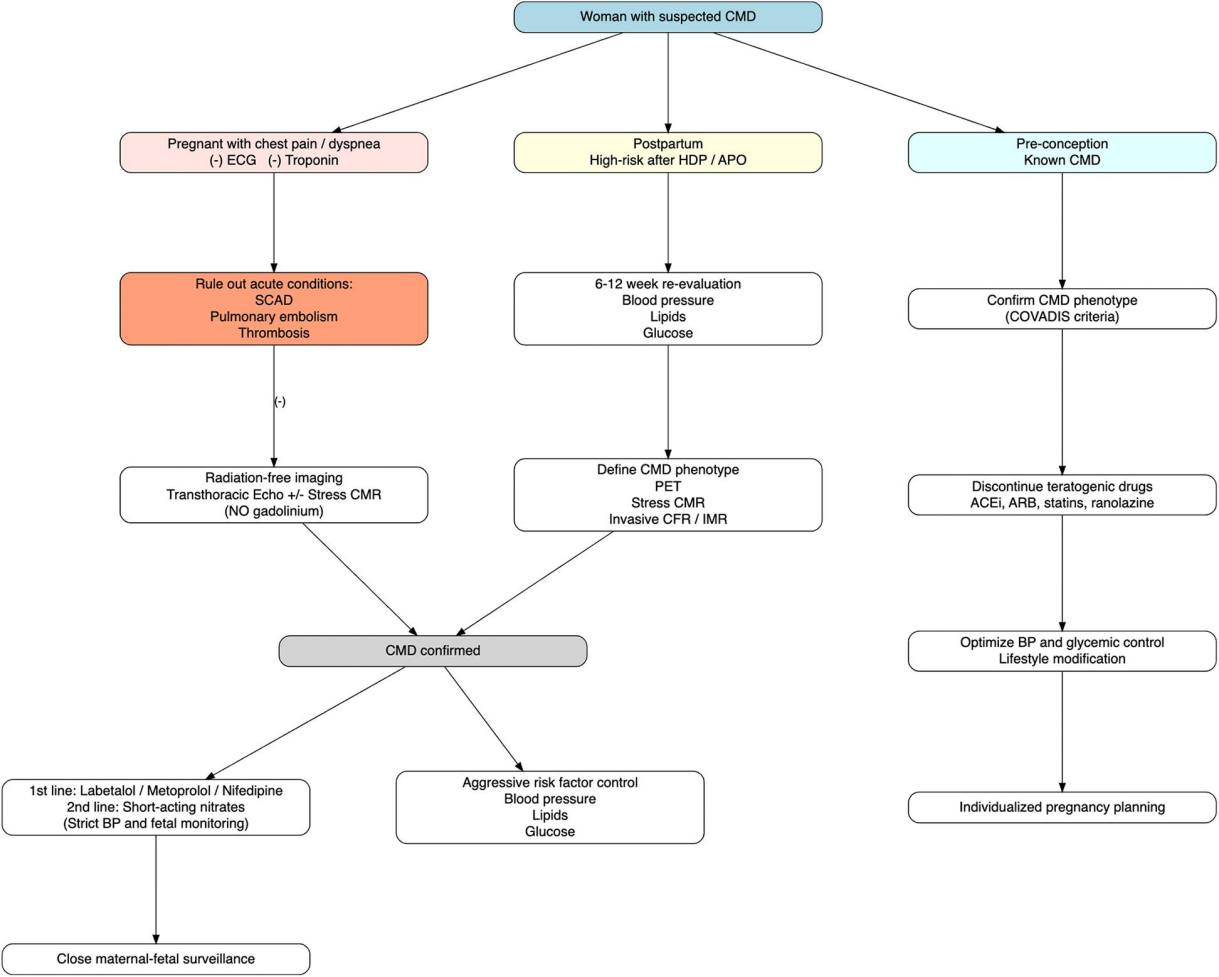
Pregnancy-stage–specific diagnostic and therapeutic algorithm for CMD, integrating pregnancy, postpartum, and pre-conception management pathways according to contemporary COVADIS/ANOCA frameworks. CMD, coronary microvascular dysfunction; HDP, hypertensive disorders of pregnancy; APO, adverse pregnancy outcomes; SCAD, spontaneous coronary artery dissection; ECG, electrocardiogram; CMR, cardiovascular magnetic resonance; PET, positron emission tomography; CFR, coronary flow reserve; IMR, index of microcirculatory resistance; ACEi, angiotensin-converting enzyme inhibitors; ARB, angiotensin receptor blockers; BP: blood pressure.

Currently, there are no established, evidence-based treatment protocols for CMD during pregnancy. Clinical management is largely empirical and extrapolated from approaches used in non-pregnant populations with modifications to prioritize both maternal and fetal safety. Therapeutic strategies should be individualized according to specific clinical settings: including the pre-conception period, throughout pregnancy and during the high-risk postpartum phase (Figure 2).

### Women with pre-existing CMD planning pregnancy

In women with known CMD who are planning pregnancy, management should focus on pre-conception counseling, medication optimization, and multidisciplinary planning. Several agents commonly used for CMD outside pregnancy, including ACE inhibitors, angiotensin receptors blockers, statins, and ranolazine are contraindicated or lack sufficient safety data during gestation and should be discontinued or replaced prior to conception. Beta-blockers with established obstetric safety profiles (e.g., labetalol, metoprolol) and calcium-channel blockers may be maintained if clinically indicated. Pre-conception counseling should address the risks of symptom exacerbation, hypertensive complications, and potential fetal effects, allowing individualized pregnancy planning and anticipatory management.

### CMD or INOCA during pregnancy

During pregnancy, management focuses primarily on symptom control, blood pressure regulation, and avoidance of maternal ischemia while preserving uteroplacental perfusion.

Beta-blockers, particularly labetalol and metoprolol, represent first-line agents in pregnant women with CMD complicated by hypertension or angina, give their established safety and efficacy in reducing myocardial oxygen demand. Calcium channel blockers, such as nifedipine widely used in obstetric practice for hypertensive disorders, may provide additional benefit by reducing vasospasm associated with CMD. Short-acting nitrates may be considered for acute symptom relief in selected cases; however, available evidence is limited to small case series and their use is often limited by the risk of maternal hypotension, reflex tachycardia and potential reduction in uteroplacental perfusion as well as the development of nitrate tolerance (11, 25).

Low-dose aspirin is routinely recommended for the prevention of preeclampsia in high-risk pregnancies, and while its direct efficacy in CMD remains unproven, its antiplatelet and endothelial-supportive effects may offer theoretical benefit. Crucially, in pregnant women presenting with chest pain or ischemic symptoms, clinicians must maintain a high index of suspicion for alternative diagnoses, including spontaneous coronary artery dissection, coronary thrombosis, or pulmonary embolism, which occur more frequently in pregnancy and the early postpartum period than true CMD. Failure to distinguish these entities may result in inappropriate management.

### Postpartum CMD after hypertensive disorders of pregnancy or APOs

In the postpartum period, particularly among survivors of hypertensive disorders of pregnancy and other APOs, management should shift toward long-term cardiovascular risk reduction and selective CMD phenotyping. Integrated follow-up should include blood pressure control, lipid profiling, glycemic assessment, and lifestyle optimization. In women with persistent angina, dyspnea, or abnormal non-invasive findings, advanced CMD assessment using PET, contrast- enhanced cardiac magnetic resonance (CMR) imaging, or invasive coronary function testing may be selectively considered for more accurate phenotyping and prognostic stratification. Postpartum pharmacologic management may progressively align with standard CMR therapies used outside pregnancy, including re-introduction of ACE inhibitors, statins, and ranolazine when no longer contraindicated, guided by breastfeeding status and overall cardiovascular risk.

### Unmet need for evidence

Lifestyle modification and optimization of metabolic factors such as weight control, glycemic control, and physical activity are key components of long-term CMD management, although data supporting these strategies in pregnant populations are sparse. Overall, the marked restrictions on pharmacologic options during gestation, combined with the paucity of interventional trials in pregnant and postpartum women with CMD, underscore the urgent need for dedicated pregnancy-specific clinical trials and mechanistic studies to guide safe and effective therapies in this vulnerable population (26, 27).

## Discussion

### Gaps in knowledge

Despite growing recognition of CMD as a key contributor to adverse cardiovascular outcomes in women, several important gaps remain in our understanding of its occurrence during and after pregnancy. First, the optimal timing and methodology for CMD assessment in this population remain unclear. Existing studies are cross-sectional, limiting insight into the temporal evolution of CMD from pregnancy through the postpartum period (8, 11). Longitudinal designs are needed to evaluate the natural history and reversibility of CMD from pregnancy through the postpartum period and determine whether CMD represents a persistent pathological process. Furthermore, diagnostic heterogeneity across studies, ranging from CFR assessment by PET and echocardiography to invasive coronary testing, introduces variability that hinders comparability and the establishment of standardized diagnostic criteria. Non-invasive modalities for CMD assessment, including stress echocardiography, CMR and PET, interrogate different components of microvascular physiology and exhibit variable sensitivity for distinct CMD endotypes. In particular, techniques primarily assessing endothelial dysfunction predominates, such as after hypertensive disorders of pregnancy (28). Establishing validated approaches to CMD assessment is essential to advance clinical understanding and inform postpartum cardiovascular risk stratification.

Importantly, diagnostic strategies cannot be applied uniformly across all reproductive stages. In symptomatic pregnant women, CMD evaluation is limited by concerns about radiation exposure and the safety of pharmacologic stress agents, making radionuclide-based techniques such as PET and CT generally inappropriate. In this setting, assessment should rely mainly on radiation-free modalities, including transthoracic Doppler echocardiography and when, feasible, CMR without gadolinium. In contrast, in the post-partum high-risk population, particularly among women with history of hypertensive disorders of pregnancy, advanced imaging techniques including PET, contrast-enhanced CMR, and even invasive coronary function testing may be considered for more accurate CMD phenotyping and long-term cardiovascular risk stratification. Finally, in the pre-conception setting, CMD assessment in women with known microvascular disease may support individualized pregnancy risk counseling and anticipatory management. Failure to clearly distinguish among these clinical scenarios may contribute to inconsistent findings across studies and limits the translation of CMD diagnostics into routine obstetric and cardiovascular care (29).

Another critical limitation lies in the inadequate control for confounding factors that may influence microvascular function. Common metabolic comorbidities such as obesity, diabetes and pre-existing cardiovascular risk often overlap with hypertensive disorders of pregnancy, making it difficult to isolate the independent impact of pregnancy-related vascular stress on coronary microcirculation. Mechanistic studies are also sparse. Although, several studies have proposed roles for anti-angiogenic factors [sFlt-1, placental growth factor (PIGF) imbalance], oxidative stress, and endothelial dysfunction, direct evidence linking these pathways to coronary microvascular alterations during pregnancy remains limited (8, 30).

Finally, few studies have explored the clinical significance of CMD in pregnancy or the postpartum period beyond subclinical markers (31, 32). The relationship between CMD and adverse cardiovascular outcomes, such as myocardial infarction, heart failure or long-term atherosclerotic disease, remains poorly defined. Therefore, postpartum follow-up strategies are crucial to identify women at high risk for persistent CMD and future cardiovascular events. Moreover, interventional research in this area is virtually absent. There is lack of trials evaluating pharmacological and non-pharmacological interventional strategies to prevent or reverse CMD in women affected by hypertensive disorders of pregnancy or other pregnancy-related cardiovascular complications. In this context, circulating biomarkers associated with CMD ay hold important therapeutic implications. Biomarkers reflecting endothelial dysfunction, inflammation, oxidative stress, and impaired nitric oxide bioavailability may not only improve CMD phenotyping and risk stratification but also help to identify novel therapeutic targets and guide individualized treatment strategies (33). However, the clinical implication of biomarker-guided therapy in pregnancy-related CMD remains largely unexplored. Addressing these evidence gaps through standardized diagnostic protocols, longitudinal mechanistic studies, and targeted interventions will be essential to clarify the natural history CMD in pregnancy and inform strategies for early prevention and treatment in this high-risk population.

### Clinical perspectives

From a clinical standpoint, recognition of CMD in pregnancy and the postpartum dysfunction carries significant implications for women's cardiovascular care. CMD may represent an early and potentially reversible manifestation of vascular injury in women with hypertensive disorders of pregnancy or other pregnancy-related complications, offering an opportunity for earlier intervention before the onset of overt cardiovascular disease. Incorporating microvascular assessment into postpartum cardiovascular follow-up could improve risk stratification, particularly in women with persistent symptoms such as chest pain or dyspnea despite normal epicardial coronaries. However, given the current lack of standardized diagnostic pathways and validated therapeutic strategies, management remains largely supportive and focused on traditional cardiovascular risk reduction. Current guidelines from the American College of Obstetricians and Gynecologists (ACOG) recommend aggressive screening for hypertension and diabetes after a pregnancy complicated by preeclampsia (34). Future integration of CMD evaluation into obstetric and cardiovascular practice along with collaborative care models bridging maternal-fetal medicine and cardiovascular specialties may enable earlier detection, targeted prevention and improved long-term outcomes for this vulnerable population.

## References

[1] Scarsini R Campo G DI Serafino L Zanon S Rubino F Monizzi G #Fullphysiology: a systematic step-by-step guide to implement intracoronary physiology in daily practice. Minerva Cardiol Angiol. (2023) 71(5):504–14. 10.23736/S2724-5683.23.06414-1 37712217

[2] Park K Wei J Minissian M Bairey Merz CN Pepine CJ. Adverse pregnancy conditions, infertility, and future cardiovascular risk: implications for mother and child. Cardiovasc Drugs Ther. (2015) 29:391–401. 10.1007/s10557-015-6597-2 26037616 PMC4758514

[3] Westbrook RH Dusheiko G Williamson C. Pregnancy and liver disease. J Hepatol. (2016) 64(4):933–45. 10.1016/j.jhep.2015.11.030 26658682

[4] Lopes van Balen VA van Gansewinkel TAG de Haas S Van Kuijk SM Van Drongelen J Ghossein-Doha C Physiological adaptation of endothelial function to pregnancy: systematic review and meta-analysis. Ultrasound Obstet Gynecol. (2017) 50(6):697–708. 10.1002/uog.17431 28170124

[5] Duvekot JJ Cheriex EC Pieters FA Menheere PP Peeters LL. Early pregnancy changes in hemodynamics and volume homeostasis are consecutive adjustments triggered by a primary fall in systemic vascular tone. Am J Obstet Gynecol. (1993) 169(6):1382–92. 10.1016/0002-9378(93)90405-8 8267033

[6] Park K Quesada O Cook-Wiens G Wei J Minissian M Handberg EM Adverse pregnancy outcomes are associated with reduced coronary flow reserve in women with signs and symptoms of ischemia without obstructive coronary artery disease: a report from the women’s ischemia syndrome evaluation-coronary vascular dysfunction study. J Womens Health (Larchmt). (2020) 29(4):487–92. 10.1089/jwh.2019.7925 31859580 PMC7194309

[7] Dvorak HF. Vascular permeability factor/vascular endothelial growth factor: a critical cytokine in tumor angiogenesis and a potential target for diagnosis and therapy. J Clin Oncol. (2002) 20:4368–80. 10.1200/JCO.2002.10.088 12409337

[8] Honigberg MC Economy KE Pabón MA Wang X Castro C Brown JM Coronary microvascular function following severe preeclampsia. Hypertension. (2024) 81(6):1272–84. 10.1161/HYPERTENSIONAHA.124.22905 38563161 PMC11096023

[9] Lane-Cordova AD Khan SS Grobman WA Greenland P Shah SJ. Long-Term cardiovascular risks associated with adverse pregnancy outcomes: jACC review topic of the week. J Am Coll Cardiol. (2019) 73(16):2106–16. 10.1016/j.jacc.2018.12.092 31023435

[10] van den Bosch AE Ruys TPE Roos-Hesselink JW. Use and impact of cardiac medication during pregnancy. Future Cardiol. (2015) 11:89–100. 10.2217/fca.14.68 25606705

[11] Pacheco C Wei J Minissian M Shufelt CL Kilpatrick SJ Quesada O Cardiovascular and pregnancy outcomes in women with coronary microvascular dysfunction: a case series. Eur Heart J Case Rep. (2019) 3(2):ytz071. 10.1093/ehjcr/ytz071 31449628 PMC6601184

[12] Enewold L Brinton LA McGlynn KA Zahm SH Potter JF Zhu K. Oral contraceptive use among women in the military and the general U.S. population. J Womens Health (Larchmt). (2010) 19:839–45. 10.1089/jwh.2009.1706 20350205 PMC2940458

[13] Merz CN Johnson BD Berga S Braunstein G Reis SE Bittner V. Past oral contraceptive use and angiographic coronary artery disease in postmenopausal women: data from the national heart, lung, and blood institute-sponsored women’s ischemia syndrome evaluation. Fertil Steril. (2006) 85:1425–31. 10.1016/j.fertnstert.2006.01.009 16600235

[14] Martin JA Hamilton BE Osterman MJ Driscoll AK Drake P. Births: final data for 2016. National center for health statistics. Natl Vital Stat Rep. (2018) 67:1–55. 29775434

[15] Wilcox AJ Weinberg CR Connor O Baird JF Schlatterer DD Canfield JP Incidence of early loss of pregnancy. N Engl J Med. (1988) 319:189–94. 10.1056/NEJM198807283190401 3393170

[16] Cameron IT Campbell S. Nitric oxide in the endometrium. Hum Reprod Update. (1998) 4(5):565–9. 10.1093/humupd/4.5.565 10027610

[17] Kulandavelu S Whiteley KJ Qu D Mu J Bainbridge SA Adamson SL. Endothelial nitric oxide synthase deficiency reduces uterine blood flow, spiral artery elongation, and placental oxygenation in pregnant mice. Hypertension. (2012) 60:231–8. 10.1161/HYPERTENSIONAHA.111.187559 22615111

[18] Mohandas R Segal MS Huo T Handberg EM Petersen JW Johnson BD Renal function and coronary microvascular dysfunction in women with symptoms/signs of ischemia. PLoS One. (2015) 10(5):e0125374. 10.1371/journal.pone.0125374 25951606 PMC4423851

[19] Suhrs HE Kristensen AM Rask AB Michelsen MM Frestad D Mygind ND Coronary microvascular dysfunction is not associated with a history of reproductive risk factors in women with angina pectoris-an iPOWER substudy. Maturitas. (2018) 107:110–5. 10.1016/j.maturitas.2017.07.004 28807722

[20] Björkman S Lilliecreutz C Bladh M Strömberg T Östgren CJ Mahmoud A Microvascular dysfunction in women with a history of hypertensive disorders of pregnancy: a population-based retrospective cohort study. BJOG. (2024) 131(4):433–43. 10.1111/1471-0528.17665 37732494

[21] Chambers JC Fusi L Malik IS Haskard DO De Swiet M Kooner JS. Association of maternal endothelial dysfunction with preeclampsia. JAMA. (2001) 285:1607–12. 10.1001/jama.285.12.1607 11268269

[22] AlBadri A Mavromatis K Bairey Merz CN. The role of coronary reactivity testing in women with no obstructive coronary artery disease. Curr Opin Cardiol. (2019) 34(6):656–62. 10.1097/HCO.0000000000000682 31490202 PMC7156026

[23] Taqueti VR Di Carli MF. Coronary microvascular disease pathogenic mechanisms and therapeutic options: jACC state-of-the-art review. J Am Coll Cardiol. (2018) 72(21):2625–41. 10.1016/j.jacc.2018.09.042 30466521 PMC6296779

[24] Taqueti VR Solomon SD Shah AM Desai AS Groarke JD Osborne MT Coronary microvascular dysfunction and future risk of heart failure with preserved ejection fraction. Eur Heart J. (2018) 39(10):840–9. 10.1093/eurheartj/ehx721 29293969 PMC5939665

[25] Yong J Tian J Yang X Xing H He Y Song X. Effects of oral drugs on coronary microvascular function in patients without significant stenosis of epicardial coronary arteries: a systematic review and meta-analysis of coronary flow reserve. Front Cardiovasc Med. (2020) 7:580419. 10.3389/fcvm.2020.580419 33195465 PMC7661556

[26] Khandkar C Rehan R Ravindran J Yong A. An updated review on therapeutic strategies in coronary microvascular dysfunction. Int J Cardiol. (2025) 428:133128. 10.1016/j.ijcard.2025.133128 40068789

[27] Kofler T Hess S Moccetti F Pepine CJ Attinger A Wolfrum M Efficacy of ranolazine for treatment of coronary microvascular dysfunction-A systematic review and meta-analysis of randomized trials. CJC Open. (2020) 3(1):101–8. 10.1016/j.cjco.2020.09.005 33458636 PMC7801206

[28] Bergamaschi L De Vita A Villano A Tremamunno S Armillotta M Angeli F Non-invasive imaging assessment in angina with non-obstructive coronary arteries (ANOCA). Curr Probl Cardiol. (2025) 50(5):103021. 10.1016/j.cpcardiol.2025.103021 40015352

[29] Almeida AG Grapsa J Gimelli A Bucciarelli-Ducci C Gerber B Ajmone-Marsan N Cardiovascular multimodality imaging in women: a scientific statement of the European association of cardiovascular imaging of the European Society of Cardiology. Eur Heart J Cardiovasc Imaging. (2024) 25(4):e116–36. 10.1093/ehjci/jeae013 38198766

[30] Lecarpentier É Vieillefosse S Haddad B Fournier T Leguy MC Guibourdenche J Placental growth factor (PlGF) and sFlt-1 during pregnancy: physiology, assay and interest in preeclampsia. Ann Biol Clin (Paris). (2016) 74(3):259–67. (English). 10.1684/abc.2016.1158 27237799

[31] Benschop L Schalekamp-Timmermans S Roeters van Lennep JE Jaddoe VW Wong TY Cheung CY Gestational hypertensive disorders and retinal microvasculature: the generation R study. BMC Med. (2017) 15:153. 10.1186/s12916-017-0917-2 28803548 PMC5554975

[32] Countouris ME Catov JM Zhu J de Jong N Brands J Chen X Association of hypertensive disorders of pregnancy with coronary microvascular dysfunction 8 to 10 years after delivery. Circ Cardiovasc Imaging. (2024) 17(5):e016561. 10.1161/CIRCIMA 38771901 PMC11115371

[33] Quarta R Martino G Romano LR Lopes G Greco FF Spaccarotella CAM The role of circulating biomarkers in patients with coronary microvascular disease. Biomolecules. (2025) 15(2):177. 10.3390/biom15020177 40001480 PMC11853534

[34] Committee on Practice Bulletins—Obstetrics. Practice bulletin No. 137: gestational diabetes mellitus. Obstet Gynecol. (2013) 122(2 Pt 1):406–16. 10.1097/AOG.0b013e31829cdbeb 23969827

